# A Unique Regulation Region in the 3′ UTR of HLA-G with a Promising Potential

**DOI:** 10.3390/ijms21030900

**Published:** 2020-01-30

**Authors:** Adi Reches, Orit Berhani, Ofer Mandelboim

**Affiliations:** The Concern Foundation Laboratories at the Lautenberg Center for Immunology and Cancer Research, The Biomedical Research Institute Israel Canada of the Faculty of Medicine, The Hebrew University Hadassah Medical School, The Hebrew University of Jerusalem, 9112001 Jerusalem, Israel; adi.reches@mail.huji.ac.il (A.R.);

**Keywords:** HLA-G, RNA binding proteins, microRNA, 3′ UTR

## Abstract

Human leukocyte antigen G (HLA-G) is a non-classical human leukocyte antigen (HLA) class I protein that interacts with inhibitory receptors and is commonly overexpressed in various cancers, thereby establishing itself as an inhibitory checkpoint immune ligand. It is also expressed in trophoblast cells during pregnancy and protects the fetus from immune rejection. Despite its crucial role and its intriguing expression pattern, the regulation of HLA-G’s expression is only partially understood. HLA-G’s mRNA is expressed in many tissues but the protein expression is restricted only to the cells mentioned above. Therefore, we suggest that HLA-G is post-transcriptionally regulated. Here, we reveal a distinctive site present only in the 3′ Untranslated region (UTR) of HLA-G, which might explain its unique expression pattern. Consequently, we attempted to find binding factors such as RNA binding proteins (RBPs) and microRNAS (miRs) that regulate HLA-G expression by interacting with this distinct site present in its 3′ UTR. Our research indicates that this site should be further studied in order to reveal its significance.

## 1. Introduction

HLA-G belongs to the family of HLA class I (also known as Major Histocompatibility Complex (MHC) class I), which presents peptides to T cells and, thereby, activates cytotoxic T cells (CTLs) of the adaptive immune system. HLA class I proteins, including HLA-G, are also ligands of NK cell inhibitory receptors. Thus, HLA class I proteins have a dual role: they activate the adaptive immune response and inhibit innate cell activity [1]. This constitutes their complex and important role of tuning the immune system. The HLA class I family can be divided into classical and non-classical groups. In humans, the classical proteins are HLA-A, -B, and -C, and each harbor high polymorphisms and are generally expressed in most tissues. The non-classical proteins are significantly less polymorphic and some of them, such as HLA-G, maintain a more tissue-specific expression pattern [2]. 

HLA-G is a crucial immune modulating molecule. It serves as an inhibitory checkpoint ligand by binding to inhibitory receptors [3] such as Leukocyte immunoglobulin-like receptor subfamily B member 1 and 2 (LILRB1 and LILRB2), and Killer cell immunoglobulin-like receptor 2DL4 (KIR2DL4) [4]. These receptors are expressed on antigen-presenting cells, NK cells, and T cells [5]. HLA-G has a unique expression pattern as it is upregulated in many types of cancers [6], whereas in normal tissues, its expression is restricted mainly to the extravillous cytotrophoblasts (EVT) of the placenta [7]. The EVT expression of HLA-G is thought to provide immune protection to the semi-allogeneic embryo, especially from attack by decidual NK cells [8]. These NK cells constitute the major lymphocyte population at the fetal–maternal interface during early pregnancy and support developmental processes such as fetal growth during pregnancy [9,10]. Besides their role in fetal protection, NK cells are innate lymphocytes best known for their ability to discriminate between self and altered-self, thus killing virally infected, transformed, and damaged cells [11,12]. Their activity is governed by integrating signals derived from a panel of activating and inhibitory receptors [11,12].

Despite the importance of HLA-G, its unique expression pattern regulation is not fully understood. The mRNA of HLA-G is expressed in several tissues however, protein expression in these tissues cannot be detected [13,14]. Thus, we speculated that HLA-G expression is heavily regulated post-transcriptionally. This regulation can be conducted by RBPs and miRs. RBPs play a part in every aspect of RNA biogenesis, including: transcription, pre-mRNA splicing, polyadenylation, RNA modification, transport, localization, translation, turnover, and immune activities [15,16,17,18,19]. miRs are small non-coding RNA molecules that usually negatively regulate genes [20]. They function via base-pairing with sequences in the target mRNA, triggering the cleavage of the mRNA or affecting the transcript’s translation efficiency. Both RBPs and miRs often bind a major regulatory site of the mRNA, the 3′ UTR [20,21]. The 3′ UTR contains binding sites for regulatory factors that fine tune the gene’s protein synthesis [22].

Six miRs were already reported to regulate HLA-G expression by binding its 3’ UTR: miR-148a, miR-148b, miR-152, miR-133a, miR-628-5p, and miR-548q [23]. However, not one of these miRs target HLA-G specifically, but rather also target other members of the HLA class I family. Regarding RBPs, we recently demonstrated that the expression of HLA-G and other classical HLA class I proteins are regulated by the RBP HNRNPR, which binds to the 3′ UTR of the transcripts and stabilizes them [24]. To the best of our knowledge, no RBP has been reported to specifically regulate HLA-G. Here, we identified a unique site in the 3′ UTR of HLA-G that is not found in the 3′ UTRs of classical HLA class I molecules. Consequently, we investigated whether there are post-transcriptional regulators which interact with this site.

## 2. Results

### 2.1. Screening for RBPs That Bind the Unique Region in the 3′ UTR of HLA-G

**Figure 1:**
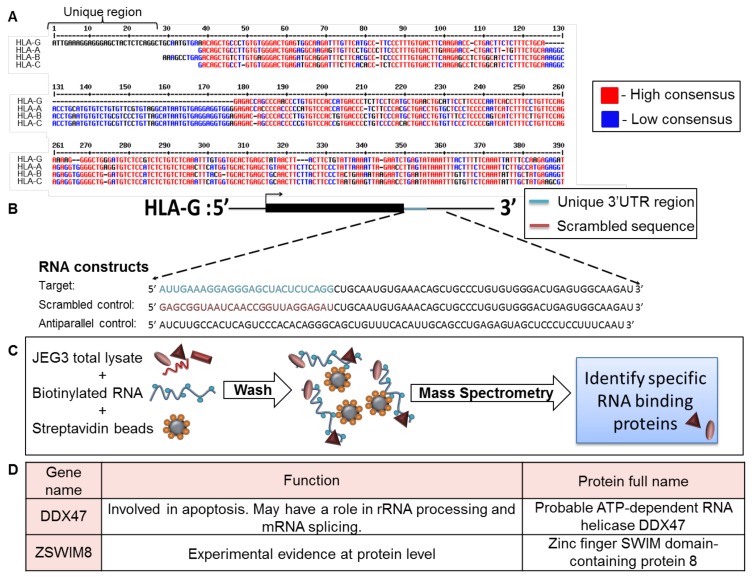
Identification of RBPs that interacted with the unique part of HLA-G 3′ UTR. (**A**) Sequence alignment of the 3′ UTRs of HLA-G, -A, -B, and –C (Reference Sequences code respectively: NM_002127.5, LC257690.1, BC091497.1, MN848254.1). The alignment was performed using the MultAlin bioinformatics tool [25]. Identical nucleotides are indicated in red. Blue nucleotides represent similarities. (**B**) Schematic representation of the RNA constructs used in the RNA affinity purification assay. (**C**) Schematic representation of the RNA affinity purification assay. (**D**) Top two identified RBP candidates.

To identify factors that specifically affect HLA-G expression, we compared the 3′ UTR of HLA-G to those of HLA-A, -B, and -C (classical HLA class I, Figure 1A). Although the HLA-G expression pattern is completely different than classical HLA class I proteins, the 3′ UTR sequence of HLA-G was similar to the 3′ UTRs of the classical HLA class I proteins. However, a unique 26 bp long region, specific only to HLA-G, was identified in the beginning of HLA-G 3′ UTR (Figure 1A). 

Since RBPs have been previously shown to regulate mRNA transcripts by binding to their 3′ UTRs [21], we initially explored whether RBPs can bind this distinct 26 bp region. We transcribed three biotinylated RNA constructs in vitro as described in Figure 1B. The first construct, named target, contained the HLA-G-specific 26 bp region followed 45 bp of the 3′ UTR sequence common to the classical HLA class I proteins (Figure 1B). We added the extra 45 bp to accommodate RBPs that may require both the unique and common regions for binding. The second and third constructs shown in Figure 1B were used as negative controls: a scrambled RNA sequence (scrambled control, Figure 1B), and an RNA sequence antiparallel to the target RNA construct (antiparallel control, Figure 1B). The stability and size of constructs was verified in gel electroporation (Figure S1). We used all three RNA constructs in an RNA affinity purification assay described in Figure 1C. In short, we immobilized the constructs on streptavidin beads and then incubated them with cytoplasmic lysates derived from the HLA-G positive cell line, JEG-3 [26] (Figure 1C). The bound proteins were eluted and sent to mass spectrometry analysis for identification. Two candidate proteins were chosen from the mass spectrometry results, which contained approximately 2000 proteins, based on their specific binding to the target construct, statistical significance score, and known function (Figure 1D). The first candidate chosen was DDX47, a helicase known to be involved in apoptosis and to bind RNA through its one annotated DEAD box site [27]. The second candidate was ZSWIM8, an unexplored zinc finger protein. Zinc fingers have a wide range of functions, from DNA or RNA binding to protein-protein interactions and membrane association [27].

### 2.2. DDX47 and ZSWIM8 RBPs Do Not Alter HLA-G Expression

**Figure 2:**
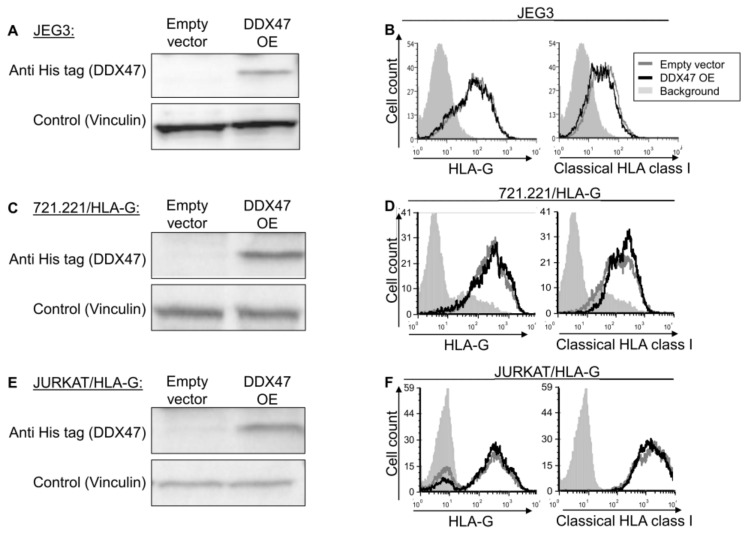
DDX47 does not influence HLA-G expression in cells expressing the HLA-G protein. (**A**,**C**,**E**) His-tagged DDX47 was overexpressed (OE) in JEG3 (**A**), 721.221 overexpressing HLA-G (**C**), JURKAT overexpressing HLA-G (**E**). Western blots were performed with anti-His-tag specific mAb and expression was compared to cells expressing an empty vector. Vinculin was used as a loading control. (**B**,**D**,**F**) Flow cytometry analysis of HLA-G (left) or classical HLA class I (right) expression on JEG3 (**B**), 721.221 overexpressing HLA-G (**D**), and JURKAT overexpressing HLA-G (**F**). Cells overexpressing His-tagged DDX47 or empty vector are represented by black and gray histograms, respectively. The filled gray histogram represents staining of cells with secondary mAb only. The figure shows one representative experiment out of three performed.

To examine whether the identified RBPs indeed regulate HLA-G, we overexpressed (OE) a His-tagged version of DDX47 in JEG-3 cells, which endogenously express HLA-G at the protein level [26] and verified it using Western blot assay (Figure 2A). HLA-G expression and classical HLA class I expression did not change upon DDX47 overexpression (Figure 2B). To test whether our observation was due to cell type (JEG3), we overexpressed DDX47 in two additional cell lines, JURKAT and 721.221 (Figure 2C,E), in which we also overexpressed HLA-G. No changes were observed in these cells (Figure 2D,F). 

**Figure 3:**
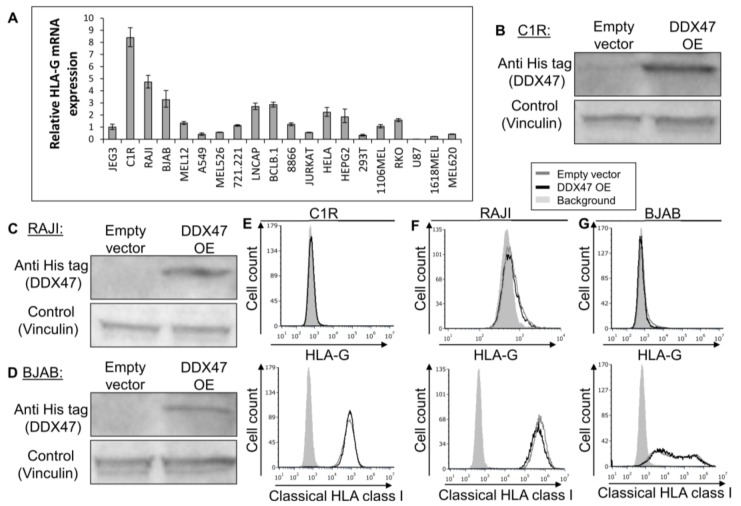
DDX47 does not induce HLA-G protein expression in cells expressing HLA-G at the mRNA level only. (**A**) HLA-G mRNA expression levels in various cell lines was evaluated using qRT-PCR. The figure shows fold enrichment of HLA-G in the indicated cell lines compared to hGAPDH. JEG3 cell line was set to 1. Shown are means ± Standard error of the mean (SEM) of triplicates. Figure shows one representative experiment out of three performed. (**B**,**C**,**D**) His-tagged DDX47 RBP was overexpressed (OE) in C1R (**B**), RAJI (**C**), and BJAB (**D**). Western blots were performed with anti-His-tag specific mAb and expression was compared with cells expressing empty vector. Vinculin was used as a loading control. (**E**,**F**,**G**) Flow cytometry analysis of HLA-G (upper panel) or classical HLA class I (lower panel) expression on C1R (**E**), RAJI (**F**), and BJAB (**G**) cells overexpressing His-tagged DDX47 or empty vector (black and gray histograms, respectively). The filled gray histogram represents staining of cells with secondary mAb only.

To further examine the regulatory role of DDX47, we screened for cells that do not express the HLA-G protein, but still express HLA-G mRNA, as detected by qRT-PCR (Figure 3A). We selected the C1R, RAJI, and BJAB cell lines since they expressed the highest levels of HLA-G mRNA (Figure 3A) and subsequently proceeded to overexpress DDX47 in these cells. We verified DDX47 expression using Western Blot (Figure 3B-D). Overexpression of DDX47 in all three cell lines did not induce the expression of the HLA-G protein (Figure 3E–G, upper panel) and no change in HLA class I expression was observed (Figure 3E–G, lower panel).

**Figure 4:**
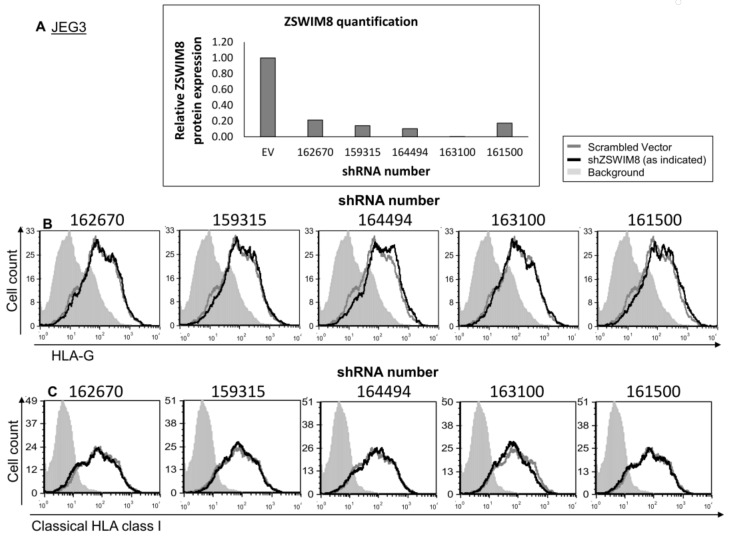
ZSWIM8 does not influence HLA-G expression. (**A**) KD of ZSWIM8 in JEG3 cells was performed using specific shRNAs (numbers indicate the shRNA catalog number). Western blots were performed with an anti-ZSWIM8 specific mAb. JEG3 cells expressing scrambled shRNA ZSWIM8 expression were set to 1. hGAPDH was used for loading control normalization. (**B**,**C**) Flow cytometry analysis of the expression of HLA-G (**B**) or classical HLA class I (**C**) on JEG3 cells transduced with the indicated shRNAs against ZSWIM8 (black histograms) compared to scrambled shRNA (gray histograms). The filled gray histogram represents the staining of scrambled shRNA with secondary mAb only. The background of the shRNA was similar to the scrambled shRNA and is not shown in the figure. Figures show one representative experiment out of three performed.

ZSWIM8, the second RBP candidate for HLA-G regulation, has a 5646 bp coding sequence (CDS) [27]. Due to its long transcript, we failed to clone it into an expression vector. Therefore, to test whether ZSWIM8 affects HLA-G expression, we knocked down (KD) its expression using five different shRNA constructs. The KD was verified using Western blot (quantified in Figure 4A). No changes were observed either in HLA-G or classical HLA class I expression (Figure 4B,C, respectively).

### 2.3. miR-1301 Is Not Involved in HLA-G Regulation

**Figure 5:**
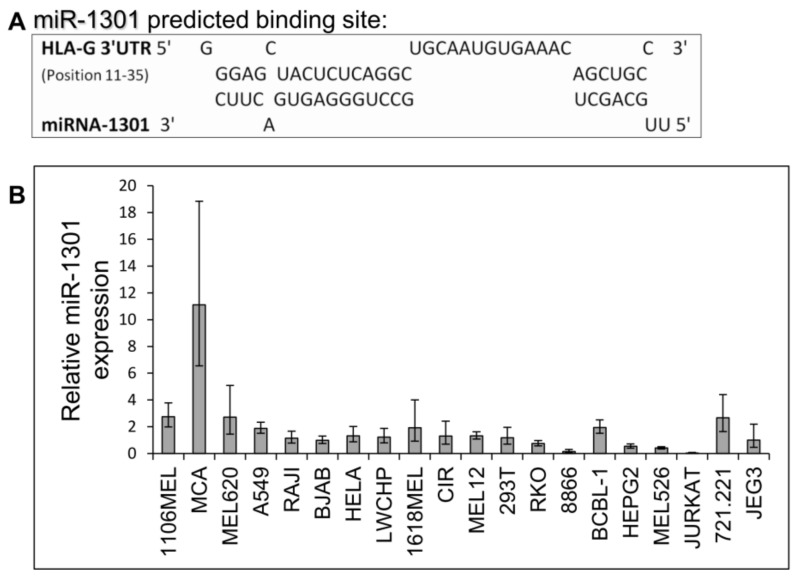
miR-1301 has a predicted binding site in the unique part of HLA-G 3′ UTR. (**A**) Predicted binding site between the unique part of HLA-G 3′ UTR (top) and hsa-miR-1301-3p (bottwm) using RNAhybrid bioinformatics tool [30]. (**B**) hsa-miR-1301-3p expression levels in various cell lines was evaluated using qRT-PCR. The figure shows fold enrichment of the miR in the indicated cell lines compared to hGAPDH. JEG3 cell line was set as 1. Shown are mean ± SEM of triplicates. Figure shows one representative experiment out of three performed.

**Figure 6:**
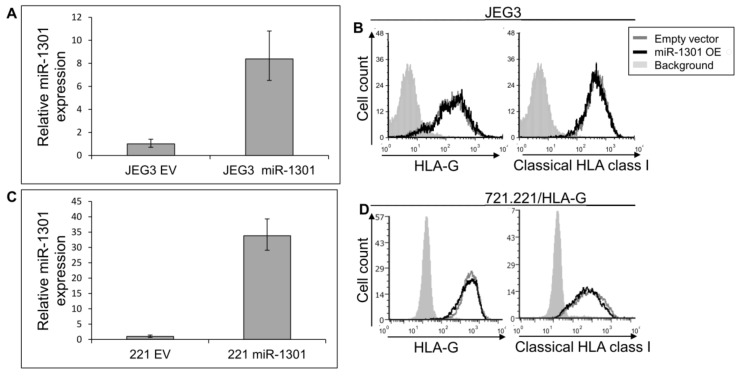
miR-1301 does not influence HLA-G expression. (**A**,**C**) hsa-miR-1301-3p was overexpressed in JEG3 (**A**) and 721.221 overexpressing HLA-G (**C**). Expression was evaluated using qRT-PCR and compared to cells expressing empty vector (EV). (**B**,**D**) Flow cytometry analysis of HLA- (left) or classical HLA class I (right) expression on JEG3 (**B**) or 721.221 overexpressing HLA-G (**D**) cells, overexpressing hsa-miR-1301-3p or empty vector (black and gray histograms, respectively). The filled gray histogram represents staining of cells with secondary mAb only. Figure shows one representative experiment out of three performed.

Next, we speculated that miRs can affect HLA-G expression by targeting its unique 26 bp region. In silico miR screening analysis identified miR-1301 to have a predicted binding site in this region (Figure 5A). miR-1301 is known to be dysregulated in early-onset preeclampsia [28], similar to HLA-G [29]. This finding strengthened our assumption that HLA-G might be regulated by miR-1301. To investigate this, we initially assayed the expression pattern of miR-1301 in various cell lines and observed similar expression levels in all cells except MCA (Figure 5B). Therefore, we chose to overexpress miR-1301 in JEG3 cells that endogenously express HLA-G [26]. Overexpression of miR-1301 was verified using qRT-PCR (Figure 6A). No alterations in HLA-G or classical HLA class I expression were detected (Figure 6B). We also overexpressed miR-1301 in 721.221 cells overexpressing HLA-G. Overexpression of miR-1301 was verified using qRT-PCR (Figure 6C), and as can be seen in Figure 6D, no changes in HLA-G or classical HLAs expressions were detected.

### 2.4. Dual Expression of miR-1301 and DDX47 Does Not Affect HLA-G Protein Expression

**Figure 7:**
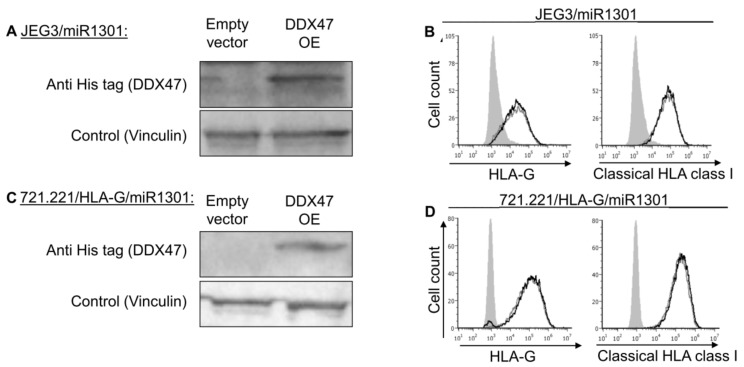
Simultaneous expression of both DDX47 and miR-1301 does not influence HLA-G protein expression. (**A**,**C**) His-tagged DDX47 was overexpressed (OE) in JEG3 (**A**) and 721.221 overexpressing HLA-G (**C**). All the cells also overexpressed miR-1301 as verified in Figure 6A,C. Western blots were performed with anti-His-tag specific mAb and expression was compared to cells expressing empty vector. Vinculin was used as a loading control. (**B**,**D**) Flow cytometry analysis of HLA-G (left) or classical HLA class I (right) expression on JEG3 (**B**) and 721.221 overexpressing HLA-G (**D**) cells overexpressing His-tagged DDX47 or empty vector (black and gray histograms, respectively). The filled gray histogram represents staining of cells with secondary mAb only. Figure shows one representative experiment out of three performed.

Many genes are often controlled by a combination of several factors. Therefore, we hypothesized that the regulation of HLA-G might require a combination of effectors. Hence, JEG3 and 721.221-HLA-G cells that overexpressed miR-1301 (Figure 6A,B) were used to overexpress DDX47 (Figure 7A,C). Even under these conditions in which both the DDX47 and miR-1301 were overexpressed, we did not observe any changes to the surface expression of either HLA-G or classical HLA class I proteins (Figure 7B,D). 

## 3. Discussion

The 3′ UTR of a given gene is an important regulatory site harboring many regulatory elements that control its post-transcription regulation [21]. The 3′ UTR of HLA-G was reported to be bound by miR-148a, miR-148b, miR-152, miR-133a, miR-628-5p, and miR-548q, all leading to downregulation of HLA-G protein expression [23]. All of them bind sites that are also present in the 3′ UTR of classical HLA class I proteins because although HLA-G has a different expression pattern, its 3′ UTR is similar to the 3′ UTRs of the classical HLA class I proteins. Thus, these miRs cannot explain why HLA-G is so uniquely expressed. 

In addition to miRs, we recently discovered and published our findings regarding the RBP HNRNPR, which binds the 3′ UTR of both HLA-G and the classical HLA class I and acts as their general positive regulator [24]. During this work, we identified a 26 bp region that is only present in the 3′ UTR of HLA-G, and is not found in any of the 3′ UTRs belonging to the classical HLA class I proteins. Accordingly, we hypothesized that this region might be involved in controlling HLA-G expression, and thus may influence its unique and specific expression pattern. 

By performing an RNA purification assay, we identified RBPs that interact with the 26 bp region. However, overexpression or downregulation of the given RBPs did not ultimately influence the surface expression levels of HLA-G. The reason for this observation might be the construct used for these assays, which included an additional 45 bp downstream sequence, common to all 3′ UTRs of classical HLA class I mRNAs. An RNA affinity purification assay capable of precipitating RBPs bound only to the 26 bp sequence may yield more specific candidates that bind this region. This assay may be difficult to perform due to the short length of the RNA transcript; it might be too small to bind streptavidin beads and RBPs simultaneously. Alternatively, maybe other RBPs that may have been precipitated but were ruled out for low statistical significance score, were those that regulate HLA-G expression specifically. We also screened for miRs with a hypothetical binding site within the 26 bp region, and selected miR-1301. We found that this miR either alone or in combination with a given RBP did not affect HLA-G surface protein expression. 

To conclude, we identified a region in the 3′ UTR of HLA-G, which is distinctive to this HLA molecule. We used different methods to identify regulators that bind to this site and specifically control HLA-G protein expression. None of our candidates affected surface HLA-G expression levels. Nevertheless, we strongly suggest that the unique site present in the 3′ UTR of HLA-G is of value and may explain the unique expression pattern of HLA-G. Hence, this site should be further studied to reveal its significance. 

## 4. Materials and Methods

### 4.1. Cell Culture

JEG3 cells were maintained in DMEM medium (Sigma-Aldrich, St. Louis, MO USA). 721.221, C1R, RAJI, BJAB and JURKAT were maintained in RPMI medium (Sigma-Aldrich). All media were supplemented with 10% fetal bovine serum and 1% MEM EAGLE, 1% penicillin-streptomycin, 1% l-glutamine, and 1% sodium pyruvate (Biological Industries, Kibbutz Beit-Haemek, Israel). No datasets were generated or analyzed during the current study.

### 4.2. RNA Affinity Purification and Mass Spectrometry

The binding of RBPs to RNA was analyzed by RNA affinity purification as previously described [31]. In short, the constructs were cloned into the pBSII plasmid. The plasmid was linearized using PspOMI restriction enzyme (Thermo Scientific (Fermentas), Waltham, MA, USA). In vitro was performed using the MEGAscript T7 transcription kit (Life Technologies, Carlsbad, CA, USA). Approximately, 10% of the Uridine-5′-triphosphate UTPs incorporated were Biotin-16-UTPs (GE Healthcare, Chicago, IL, USA). We immobilized 260 pmol of biotinylated RNAs to streptavidin sepharose beads (GE Healthcare, Chicago, USA) for 4 h. This complex was then incubated with 5 μg of cytoplasmic extracts from JEG3 cells and placed overnight at 4 °C. The cytoplasmic extract contained RNAase inhibitor (100 units/mL of extract) to prevent RNA degradation in solution. After washing the unbound lysate, the protein on the beads were eluted and sent for mass spectrometry analysis. Analysis was performed by the Smoler Proteomics Center, Haifa, Israel.

### 4.3. Generation of Lentivirus, Knockdown, Overexpression

ZSWIM8 knockdown vectors and scrambled vector cloned into pLKO.1-puro plasmids were purchased from Sigma Aldrich. Transduced JEG3 cells were grown in the presence of 2 μg/mL puromycin. DDX47 and HLA-G were cloned into the pHAGE-DsRED(−)-eGFP(+) lentiviral vector that also contains GFP. miRNA-1301 vectors were generated as previously described [32]. The oligonucleotides used to generate artificial short hairpin RNAs that function as orthologues of pre-miRNA-1301 hairpins (below) were annealed. The hairpins were then inserted into the pTendoplasmic reticulum vector. Then, the hairpins were excised from the vector with the H1 RNA polymerase III promoter and cloned into the lentiviral vector SIN18-pRLL-hEFIp-EGFP-WRPE. We generated the lentiviruses in 293 T cells using a transient three-plasmid transfection protocol as previously described [32]. Transduction efficiency into the cells was assessed by GFP expression and only cell populations with >90% efficiency were used for experiments. 

Primer sequences: miR-130 forward: gatccccTTGCAGCTGCCTGGGAGTGACTTCttcaagagaGAAGTCACTCCCAGGCAGCTGCAAtttttggaaa, miR-1301 reverse: agcttttccaaaaaTTGCAGCTGCCTGGGAGTGACTTCtctcttgaaGAAGTCACTCCCAGGCAGCTGCAAggg, DDX47 forward (NotI): atGCGGCCGCAccatggcggcaccc DDX47 reverse (AgeI): TGACCGGTTTAATGGTGATGGTGATGGTGACGGCCTTTCCG, HLA-G forward (NotI): GCGGCGGCCGCGCCGCCACCATGGTGGTCATGGCG HLA-G 14 bp deletion reverse (AgeI): GCGACCGGTAAAGTTCTCATGTCTTCCATTTA

### 4.4. RNA Extraction and cDNA Preparation 

Total RNA was extracted with TRI reagent (T9424, Sigma-Aldrich), followed by Turbo-DNase (Ambion, Hampton, VA, USA) treatment. A poly(A) tail was added using the poly(A) kit (Ambion). cDNA libraries were generated from 1 μg of RNA using M-MLV reverse transcriptase (Invitrogen, Waltham, MA, USA), in the presence of an adapter primer. Detection of the various transcripts was performed with qRT-PCR.

### 4.5. Quantitative Real-Time PCR

For qRT-PCR, cDNAs were labeled in SYBR Green based detection using a QuantStudio 12k Flex Real-time PCR cycler (Life Technologies, CA, USA). The primers used for targeting HLA-G, GAPDH, and miR-1301 were: GAPDH forward: GAGTCAACGGATTTGGTCGT, GAPDH reverse: GATCTCGCTCCTGGAAGATG, HLA-G forward: AGTCAAAGACAGGGTGGTGG, HLA-G reverse: GGAGTGGCTCCACAGATACC

The reverse primer for miR-1301 was the 3′ adapter primer (3′ RACE outer primer from the FirstChoice RLM-RACE kit (Thermo Scientific, Waltham, MA, USA), The forward primer we used was: TTGCAGCTGCCTGGGAGTGACTTC

### 4.6. Western Blot Analyses

Lysates of JEG3, 721.221, JURKAT, C1R, RAJI, or BJAB cells were prepared using lysis buffer containing SDS, PMSF, and aprotinin, followed by SDS gel electrophoresis. Proteins were transferred onto a nitrocellulose membrane and specific protein bands were detected using antibodies detecting His-tag (MCA1396, Abcam, Cambridge, UK (1:1000)), ZSWIM8 (sc-514204, Santa Cruz, CA, USA (1:1000)) Vinculin (AB-ab129002, Bio-Rad, Hercules, USA (1:1000)), and GAPDH (SC-32233, Santa Cruz, CA, USA (1:1000)). All were diluted in 5% BSA in 1× PBS. Chemiluminescence was detected by secondary antibody-linked horse-radish peroxidase (HRP, Jackson ImmunoResearch, West Grove, PA, USA).

### 4.7. Flow Cytometry

The following antibodies were used throughout the manuscript: anti-HLA-G (purchased from AbD Serotec, Hercules, USA, catalog number: MCA2044), anti-classical HLA class I proteins mAb (clone W6/32, self-produced from hybridoma). Staining was performed with 0.2 μg mAbs per 100,000 cells. Binding was detected by a secondary antibody used in a final dilution of 1:200 (Abcam, Cambridge, UK, Alexa Flour^®^ 647 catalog number 115-606-062). All stainings were analyzed by flow cytometry using the FCS Express software.

## Supplementary Materials

Supplementary materials can be found at https://www.mdpi.com/1422-0067/21/3/900/s1.
