# Supplementary material for: A Unique Regulation Region in the 3′ UTR of HLA-G with a Promising Potential
Source: Int J Mol Sci. 2020 Jan 30;21(3):900. doi: 10.3390/ijms21030900 (PMC7037441; doi:10.3390/ijms21030900)
Supplement: Supplementary file 1 [file ijms-21-00900-s001.pdf]

International Journal of
Molecular Sciences

MDPI

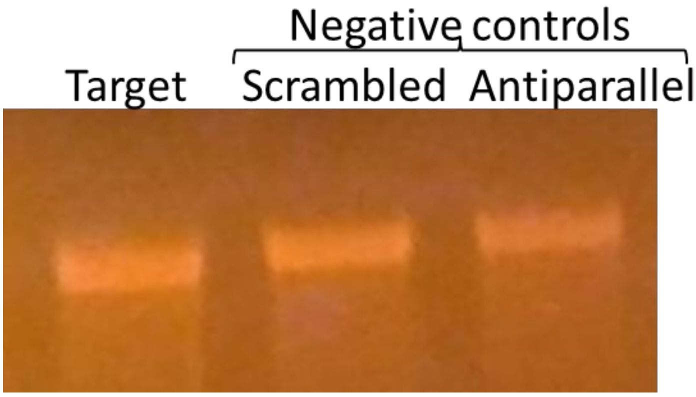

Figure S1. bands of the constructs prepared for pull down. RNA constructs bands prepared for pull down from electrophoresis gel (sequence presented in figure 1B).
